# Medical Items

**Published:** 1897-11

**Authors:** 


### *MEDICAL ITEMS.*

---

Dr. J. Scott Todd, of Atlanta, has returned from Europe.

---

Dr. Henry W. Terrell has moved from Greenville, Georgia, to LaGrange.

---

What is the appropriate flower for a diabetic patient to wear? Sweet peas.—*Ex.*

---

"Rastus," said Uncle Remus, "did yer ever take notice how things is evened up in dis world? De nigger ketches de smallpox, but de yellow fever mos' ginerally gits de white man."

---

According to a contemporary, over 500,000 French women have had their ovaries removed within the last fourteen years. And still the French are wondering at their decreasing birth-rate.

---

The Tenth Annual Meeting of the Southern Surgical and Gynecological Association will be held in St. Louis, November 9th, 10th and 11th. The Southern Hotel will be headquarters. A complete program has been prepared and a full meeting is expected.

---

"What drugs are most useful in general practice?" was recently answered by every member of Willesden Medical Society, and from the list the following are selected: Potassium iodide, Fowler's solution, tincture of digitalis, solution of strychnine, calomel, and opium.—*The Lancet, London.*

---

Every physician who uses electricity should send for a copy of The Electro-Therapeutist, a monthly journal devoted to electrotherapeutics for the general practitioner. Write the editor, Wm. F. Howe, M.D., Indianapolis, Ind., mentioning this journal, and he will send you sample copies gratis.

---

Insist upon having your prescriptions filled as written. No matter how great may be the ability of the practitioner as a diagnostician, the patient receives no advantage if the preparations administered are of indifferent quality; and if, as the result of substitution of imitation goods, the outcome of a given case is unsatisfactory, the physician will be blamed, not the druggist.

---

BRAITHWAITE'S *Retrospect of Medicine* for January, 1897, is just out, containing abstracts of the most important medical and surgical papers published in the latter part of 1896. This is really a "half-yearly journal containing a retrospective view of every discovery and practical improvement in the medical sciences." Edited by Dr. James Braithwaite, of London. Published by G. P. Putnam's Sons, New York.

---

WE desire to thank Dr. Chas. P. Noble, of Philadelphia (1637 North Broad street), for interesting reprints entitled "Observations Concerning Movable Kidney," "Ectropion of the Cervix in Nulliparæ Resembling Laceration," "New Method of Suturing the Abdominal Wall," "Contributions to the Technique of Operations for the Cure of Lacerations of the Pelvic Floor in Woman," "Vaginal Incision and Drainage of Suppurating Hematoceles Due to Ectopic Gestation," "Clinical Report on the Course of Pregnancy and Labor as Influenced by Suspensio Uteri," "The Development and Present Status of Hysterectomy for Fibromyomata," "Remarks upon the Use of the Buried Permanent Suture in Abdominal Surgery," "Report of the Kensington Hospital for Women."

---

FOR four years the Medical Society of Virginia has offered a prize of four hundred and fifty dollars for a complete History of

Medicine and Surgery in Virginia. Four manuscripts were submitted at the late meeting of the society, and the prize was awarded to Dr. Arthur Jordan, of Richmond. A committee of the society will edit the manuscripts, and from the four will prepare a book containing all available matter upon the history of medicine and surgery in the State. Dr. McGuire of Richmond, Dr. Nash of Norfolk, and Dr. Price of Philadelphia, also contributed funds to the prize. Thanks are due these gentlemen and to the Medical Society for having provided for this valuable work. Why can we not get a History of Medicine and Surgery in Georgia in a similar manner?

---

At a regular monthly meeting of the Newport Medical Society, held on August 18th, 1897, a paper previously communicated to the Rhode Island Medico-Legal Society, on August 12th, was, by request, read by Dr. H. R. Storer, Honorary President, in which it was shown that the induction of unnecessary abortion is probably now as prevalent in this country as when the subject was brought to the attention of the American Medical Association by him forty years ago, in 1857. The Society thereupon unanimously appointed a committee "to obtain through correspondence with medical societies and otherwise such action by the profession as may tend to lessen the occurrence of criminally induced abortion." The chairman of the committee, Dr. Valentine Mott Francis, Newport, Rhode Island, will be glad to receive data or suggestions from any one upon this important subject.

---

AT the late meeting of the American Academy of Medicine, a medical society supposed to possess a superior intellectual status, Dr. Albert H. Gihon, retired Medical Director U. S. Navy, stated that Dr. Benjamin Rush was the only practitioner of medicine whose name is signed to the Declaration of Independence. Before such a body as the Academy of Medicine this statement should not have remained unchallenged. The *Georgia Journal of Medicine and Surgery* has corrected the error by mentioning Lyman Hall, of Georgia, Oliver Walcott, of Connecticut, Josiah Bartlett and Mathew Thornton, of New Hampshire, all physicians, who signed that great paper. "It seems to us ridiculous that these superiorly educated beings, assembled together in the City of Brotherly Love, should exhibit so monumental an ignorance concerning the fathers of the Republic in which they live. Even the editor of the *Bulletin,* issued by the Academy, let the assertion stand without comment."

---

THE following, which we take from the pages of an esteemed contemporary, are our sentiments:

It is in order to say a few words upon a subject that is of vital importance to us and to every publisher of a medical periodical. There is an old saying, "that money makes the mare go." Now, if we do not have money, we cannot make our journal go. While medical men practice their science for the health of mankind in general, they expect at the same time to make a living by it, if not money; so it is with publishers of medical literature. They disseminate knowledge among the profession, and while they love the profession, they cannot afford to put their time and capital into a business that brings them no financial return. We are not publishing a magazine for our health's sake. We wish to get a fair return for our labor. We derive certain profit from advertisements that appear each month, but these are a side issue. We expect our support to come from our subscribers, who are the direct beneficiaries of the journal. We know that many medical men are poor, but we cannot run a business on a charitable basis, and trust to luck to pay our bills. No matter how poor a doctor may be he must, if he expects to keep up with the times, read a good medical journal. He should do this even if he has to let his grocer, butcher and landlord wait. This is a delicate matter that we do not like to discuss, and will not again unless we are forced to. At the same time we are doing only what many other publishers are at present, reminding our delinquent subscribers of their obligation to this fountain of knowledge.

---

THE wildest sort of rumors have been put in circulation by some sensation-loving idiot concerning the prevalence of small-pox and yellow fever in Atlanta. It is well known that Atlanta has freely and unconditionally welcomed all refugees from yellow fever districts. Among several hundred who have taken advantage of this offer there have been two who, having brought the infection with them, have gone through a mild attack of yellow fever here and recovered. *There has not been a case among the resident population of Atlanta.*

As to small-pox, there have been twenty-three cases, all negroes. All cases, as soon as discovered, have been taken to the pest-house, about two miles from the city. A corps of physicians, employed by the Board of Health, have just completed a thorough house-to-house vaccination, particularly among the negroes, and the city is now regarded as practically safe against further progress of small-pox.

The fool who started these reports could easily have ascertained the facts by enquiring of the Board of Health. But that would have spoiled his little story.

---

WE are just informed that the *Magazine of Medicine*, formerly the *Moody*, after its brilliant but brief career, has yielded up its gentle spirit, and thus another is added to the long list of journals that have passed in the night. The "Innovation in Current Medical Literature," which was proclaimed as the drawing card of this particular "magazine," did not draw. The most striking feature of this "innovation" appeared to be the infinite variety of matter which was offered, no doubt to satisfy the widely different tastes of its many readers. Its "departments" included a vast field of human activity; medicine (regular, homeopathic, eclectic, and quack), literature, science, insurance, railroads, household, and others which we have forgotten. Two other important "innovations" were the monthly praises of Ingersollism and the home-made poetry by the editor. We do not know which contributed more to the fatal ending.

The *Moody Magazine of Medicine* began, as others have begun, with an *I-came-I-saw-I-conquered* flourish, its especially appointed mission being to give light to the "rank and file of the profession." A part of this ambition was nobly attained. It was certainly *rank*. However, it is dead. After life's fitful fever may it sleep well. We may never look upon its like again. *Hinc illæ lacrimæ.*

---

OVER in an Alabama town, where the shotgun quarantine is in force, a physician was called to see a patient about three miles in the country. When he started home he was met by a shotgun committee and will be held in detention-camp until frost.

An interesting class are the refugees who try to convince themselves that they are not refugeeing from the plague. I was talking to a man who had been among the first to leave a certain town when the fever broke out, bringing his family with him. He had left his business to take care of itself, and yet he denied that he was a refugee.

"I don't believe there is any yellow fever in my town," he said. "This fellow Guiteras in talking through his hat."

"Why did you leave?" I asked.

"I wanted to give my family an outing," he replied glibly, "so I just packed up and came here. I have friends in Atlanta and it is very pleasant here. Oh, no; I wasn't a bit afraid. Of course, the others got a little excited. That's one reason I brought them away."

"Have many people left your town?"

"Only a certain class of them."

"What class is that?"

"Most all of those who could get away. The stay-at-home club is composed of those who couldn't."—*Atlanta Journal.*

